# Paramedics . . . Why do research?

**DOI:** 10.29045/14784726.2022.06.7.1.1

**Published:** 2022-06-01

**Authors:** Gregory Adam Whitley, Caitlin Wilson

**Affiliations:** University of Lincoln; East Midlands Ambulance Service NHS Trust ORCID iD: https://orcid.org/0000-0003-2586-6815; University of Leeds; North West Ambulance Service NHS Trust ORCID iD: https://orcid.org/0000-0002-9854-4289

Paramedics and other registered healthcare professionals choose from a variety of career pathways, broadly split into four categories: clinical practice, leadership and management, education and research (College of Paramedics, 2018). These roles do not exist in silos; instead they intertwine to form interesting and bespoke roles in a variety of settings. The choice of career pathway is highly individual, subject to personal preference and largely influenced by the sense of career achievement and satisfaction.

Scholarship is expected and valued in clinical practice, leadership and management and education, but it is research that produces discipline-specific knowledge that informs all areas of professional practice (Health Education England, 2022). Research acts as one of the foundations that underpins professional practice and therefore requires continued support to encourage and foster early career researchers, enhancing capacity and capability so that the profession can continue to develop (Trusson et al., 2019).

This article aims to illuminate four reasons why one might consider a career in research: curiosity, autonomy, satisfaction and potential for impact. We would like to make clear that all healthcare professional pathways have elements of these four reasons, and all are equally valuable. Here, we promote research specifically as an academic exercise as it is an underrepresented career pathway for paramedics (arguably the least represented).

## Curiosity

In our clinical practice we continually come across decisions, treatments and practices that we do not fully understand. We may question them and get the ‘this is what we’ve always done’ response. But as clinicians we are required by our regulatory body to have a better reason than ‘this is what we’ve always done’. We need to employ the principles of evidence-based practice (Sackett et al., 1996).

Choosing a career in research allows us not only to follow evidence-based practice but also to follow our curiosity to the boundary of knowledge and beyond, to create new knowledge and actively contribute to the evidence base. It provides the opportunity to turn those niggling questions of ‘Why? Why? Why?’ into a research project which will advance the evidence base of paramedic practice and contribute to improving patient care (Trusson et al., 2019).

## Autonomy

In clinical practice, we cannot control which patients we attend. In education, we follow a prescribed curriculum informed by organisational, professional and regulatory guidelines. In leadership and management, the strategy and direction of the organisation along with prevailing winds from government must be followed.

In research we set our own compass. We have the autonomy to choose a topic of research that we are passionate about, choose who we want to work with from around the globe and design comprehensive studies to answer complex clinical questions. We can develop new methods for undertaking research and new theories to help us understand phenomena. While there are still organisational directions and prevailing political winds to abide by, this level of autonomy is still liberating.

## Satisfaction

The sense of satisfaction experienced when achieving return of spontaneous circulation for the first time, watching your students graduate or seeing a service improvement project that you have led come to life is difficult to beat. However, we think it is fair to say that the excitement and thrill of that first publication, achieving ‘published author’ status, is exhilarating. Knowing that your published work is out there, forever, for the world to see is very exciting, and a little scary. Your first publication may not be a research article, it may be an opinion piece, a book review, a CPD article or even a book chapter, but it does stir something inside that makes you want more.

There is an element of pride with our published work, we want to share it with others, our friends and family perhaps, but more importantly there is an element of pride within, that pushes us to do better each time. There is also satisfaction in knowing that our published research will last forever and will likely impact someone somewhere long after we are gone.

## Potential for impact

Impact is an interesting concept which is difficult to define and even more difficult to measure (Greenhalgh et al., 2016; Woodcock et al., 2021). The most important type of impact, in our opinion, is that which improves patient outcomes, specifically mortality, morbidity and quality of life. As healthcare professionals, we may have direct one-to-one impact on patient outcomes through clinical practice, or indirect impact on patient outcomes through leadership and management, education or research. Research, in our opinion, has the potential to achieve significant impact through *international* changes to clinical guidelines, policy, service models and legislation/regulation.

Whether you are starting out in your career, or are an experienced healthcare professional, we would encourage you to consider a career in research. Remember, research is a career pathway not just for a select few, but for anyone who is curious and motivated.

## Acknowledgements

We would like to thank Dr Bill Lord for helpful feedback whilst preparing this article.

## Conflict of interest

Both authors are on the editorial board of the *BPJ*.
